# Thermospermine modulates expression of auxin-related genes in *Arabidopsis*

**DOI:** 10.3389/fpls.2014.00094

**Published:** 2014-03-14

**Authors:** Wurina Tong, Kaori Yoshimoto, Jun-Ichi Kakehi, Hiroyasu Motose, Masaru Niitsu, Taku Takahashi

**Affiliations:** ^1^Division of Bioscience, Graduate School of Natural Science and Technology, Okayama UniversityOkayama, Japan; ^2^Faculty of Pharmaceutical Sciences, Department of Analytical Chemistry, Josai UniversitySakado, Japan

**Keywords:** *Arabidopsis thaliana*, auxin, lateral root, polyamines, thermospermine, xylem

## Abstract

Thermospermine, a structural isomer of spermine, is widely distributed in the plant kingdom and has been shown to play a role in repressing xylem differentiation by studies of its deficient mutant, *acaulis5* (*acl5*), in *Arabidopsis*. Our results of microarray and real-time PCR analyses revealed that, in addition to a number of genes involved in xylem differentiation, genes related to auxin signaling were up-regulated in *acl5* seedlings. These genes include *MONOPTEROS*, an auxin response factor gene, which acts as a master switch for auxin-dependent procambium formation, and its target genes. Their expression was reduced by exogenous treatment with thermospermine or by transgenic induction of the *ACL5* gene. We examined the effect of synthetic polyamines on the expression of these auxin-related genes and on the vascular phenotype of *acl5*, and found that tetramines containing the NC_3_NC_3_N chain could mimic the effect of thermospermine but longer polyamines containing the same chain had little or no such effect. We also found that thermospermine had an inhibitory effect on lateral root formation in wild-type seedlings and it was mimicked by synthetic tetramines with the NC_3_NC_3_N chain. These results suggest the importance of the NC_3_NC_3_N chain of thermospermine in its action in modulating auxin signaling.

## Introduction

Polyamines such as spermidine and spermine are abundant in living cells and are involved in a variety of physiological processes including embryogenesis, growth, fruit ripening, and stress responses in plants (Kusano et al., 2007; Alcázar et al., 2010; Takahashi and Kakehi, 2010). Polyamines interact with nucleic acids and membranes and influence many enzymatic reactions. In recent years, attention has been focused on the action of polyamines on the protein synthesis machinery. There is increasing evidence that polyamines stabilize the RNA structure, promote the association of ribosomal subunits, and may affect the rate or efficiency of nascent polypeptide chain elongation (Igarashi and Kashiwagi, 2011). A structural isomer of spermine, thermospermine (Table 1), was first discovered in thermophilic bacteria and has also been implicated in the protein synthesis under extreme environmental conditions (Oshima, 2007). The *Arabidopsis acaulis5* (*acl5*) mutant is defective in the synthesis of thermospermine and shows severe dwarf phenotype, indicating that thermospermine is required for stem elongation (Kakehi et al., 2008). Since *ACL5* is predominantly expressed during xylem formation from procambial cells to differentiating xylem vessels (Clay and Nelson, 2005; Muñiz et al., 2008) and *acl5* has over-proliferated xylem vessels (Hanzawa et al., 1997), its dwarf phenotype may be primarily attributed to excess xylem differentiation and thermospermine appears to act as a repressor of xylem differentiation. While exogenous thermospermine can partially but significantly rescue the stem growth of *acl5*, spermine has no such effect (Kakehi et al., 2008). Indeed, *acl5* contains spermine produced by the action of spermine synthase, SPMS, while loss-of-function mutants of *SPMS* show wild-type phenotype under normal growth condition (Imai et al., 2004). We have found that norspermine is also able to rescue the growth of *acl5* (Kakehi et al., 2010). It is noted that the NC_3_NC_3_N (hereafter expressed as 3-3) arrangement of carbon chains is present in both thermospermine (3-3-4) and norspermine (3-3-3) but not in spermine (3-4-3), suggesting its structural significance for the biological function.

**Table 1:** **Linear polyamines used in this study**.

**Triamines**	**H_2_N(CH_2_)_a_NH(CH_2_)_b_NH_2_**
	**a-b**
Norspermidine	3-3
Spermidine	3-4
**Tetramines**	**H_2_N(CH_2_)_a_NH(CH_2_)_b_NH(CH_2_)_c_NH_2_**
	**a-b-c**
N-(2-Aminoethyl)norspermidine	3-3-2
Norspermine	3-3-3
Thermospermine	3-3-4
N-(5-Aminopentyl)norspermidine	3-3-5
Spermine	3-4-3
**Pentamines**	**H_2_N(CH_2_)_a_NH(CH_2_)_b_NH(CH_2_)_c_ NH(CH_2_)_d_NH_2_**
	**a-b-c-d**
Homocaldopentamine	3-3-3-4
N,N'-Bis(4-aminobutyl)norspermidine	4-3-3-4

Thermospermine may be widely distributed among the plant kingdom, based on the presence of putative orthologs to *ACL5* in different plant species (Knott et al., 2007; Minguet et al., 2008; Takano et al., 2012). Our analyses of suppressor mutants of *acl5* named *sac* have suggested that thermospermine enhances translation of *SAC51*, a putative basic helix-loop-helix (bHLH) transcription factor gene, by reducing the inhibitory effect of small upstream open reading frames (uORFs) located in its 5′ leader sequence (Imai et al., 2006). A study of the *thickvein* (*tkv*) mutant, which represents another allele of the *ACL5* locus, suggests that the reduction in polar auxin transport is responsible for the vascular phenotype of the mutant (Clay and Nelson, 2005). Muñiz et al. (2008) revealed that cell death occurs before the onset of secondary cell wall formation in the xylem vessels of *acl5*, suggesting a role of *ACL5* for preventing differentiating xylem vessels from premature programmed cell death (Muñiz et al., 2008). More recently, *acl5* mutants were shown to be more sensitive to pathogens than wild-type plants, suggesting a function of thermospermine in defense signaling (Marina et al., 2013). However, the precise mode of action of thermospermine remains to be clarified. For comprehensive understanding of the function of thermospermine in plants, we performed microarray and real-time RT-PCR experiments with *acl5* seedlings. Our results revealed that, in addition to a number of genes involved in xylem differentiation, genes related to auxin signaling were up-regulated in *acl5* seedlings. They were reduced by thermospermine and synthetic tetramines containing the 3-3 chain (Table 1). These tetramines are further shown to have an inhibitory effect on lateral root formation in wild-type seedlings.

## Materials and methods

### Chemicals

All polyamines used in this study are shown in Table 1. Spermidine, spermine, and norspermidine were purchased from Sigma (St. Louis, MO, USA). The other uncommon polyamines were synthesized by the published method (Niitsu et al., 1992). MS salts for plant nutrition were purchased from Wako (Osaka, Japan).

### Plant materials and growth conditions

*Arabidopsis thaliana* accession Columbia-0 (Col-0) was used as the wild type. The original *acl5-1* mutant in the accession Landsberg *erecta* (L*er*) was backcrossed more than 7 times into Col-0 (Hanzawa et al., 2000). *spms-1* in the Col-0 background has been described previously (Imai et al., 2004). Transgenic *acl5-1* lines carrying the *HS-ACL5* construct and the *SAC51-GUS* construct have been described in Hanzawa et al. (2000) and Imai et al. (2006), respectively. In experiments with *monopteros* (*mp*) in the L*er* background (Berleth and Jürgens, 1993), which were obtained from ABRC, wild-type L*er* and *acl5-1* in L*er* were used as a reference and for crosses. Prior to germination, seeds were surface sterilized with commercial bleach (5% sodium hypochlorite) supplemented with 0.02% (v/v) Triton X-100, and rinsed three times with sterile distilled water. For microarray experiments, seeds were germinated and grown on Murashige and Skoog (MS) agar medium supplemented with 3% sucrose for 7 days under continuous light at 22°C. For the growth in the presence of polyamines, each polyamine was added to the MS agar or liquid medium at 100 μM. For treatment of seedlings with polyamines for 24 h or with heat shock at 37°C for 2 h, seeds were germinated, grown in MS solutions for 7 days under continuous light at 22°C, and incubated as described in figure legends.

### RNA extraction and real-time RT-PCR

Total RNA was isolated from whole seedlings according to the SDS-phenol method and reverse-transcribed with oligo-dT primers and PrimeScript™ reverse transcriptase (Takara, Kyoto, Japan) at 42°C for 1 h. The resulting first-strand cDNA was directly used for real-time PCR with target gene-specific primers (Table 2). PCR reactions were performed using KAPA SYBR FAST qPCR Kit (KAPA Biosystems, Woburn, MA, USA) and the DNA Engine Opticon2 System (Bio-Rad, Hercules, CA, USA). ACTIN8 (At1g49240) was used as an internal standard in the reactions. Data from three independent biological replicates each with two technical replicates are expressed as means ± *SD*.

**Table 2:** **Primer sequences of the genes used for RT-PCR analysis**.

**Gene name**	**AGI code**	**Forward primer sequence**	**Reverse primer sequence**
*ACL5*	At5g19530	ACCGTTAACCAGCGATGCTTT	CCGTTAACTCTCTCTTTGATTC
*XTH3*	At3g25050	GTGTTTTTGTAGTAACGTTATGG	GGTTGGATTGAACCAAAGCAA
*VND6*	At5g62380	ATGGAAAGTCTCGCACACAT	CTCTTCCACATAACTCTTGG
*VND7*	At1g71930	CGATGCATCAATATGGCAAC	AGGGAAGCATCCAAGAGAAT
*CNA*	At1g52150	GGTATTTGCTGATTCATGAGC	ATGGTTTACACTTGACAGAGC
*ATHB8*	At4g32880	AGCGTTTCAGCTAGCTTTTGAG	CAGTTGAGGAACATGAAGCAGA
*MP/ARF5*	At1g19850	GATGATCCATGGGAAGAGTT	TAAGATCGTTAATGCCTGCG
*TMO5L1*	At1g68810	CACCACCAAAACGGATAAAG	CGTTTTGAGACGCATAGCTT
*PIN1*	At1g73590	CTTAGCACTGCGGTGATATT	TTGCTGAGCTCCTACTTAAG
*PIN6*	At1g77110	CTATCGTACAGGCTGCTCTA	CTCCTCAAGAACAACTCTTA
*YUC2*	At4g13260	ATGTGGCTAAAGGGAGTGAA	AACTTGCCAAATCGAAACCC
*IRT1*	At4g19690	GTCTAATCACTCTAGCCATTGA	TGTATACTCAGCCTGGAGGA
*PXY/TDR*	At5g61480	CGGTTACATTGCACCAGAAT	GCTTGTACACAACAACGCAA
*SAC51*	At5g64340	AATTGCCAGGCTGAGTACTT	GACCGACCTACTATATCCTT
*ACT8*	At1g49240	GTGAGCCAGATCTTCATTCGTC	TCTCTTGCTCGTAGTCGACAG

### Microarray analysis

Microarray experiments were performed in three independent biological replicates using the Agilent *Arabidopsis* 3 oligo microarray (Agilent Technologies, Wilmington, DE, USA) in Hokkaido System Science (Sapporo, Japan). RNA quality was confirmed by gel electrophoresis as well as by OD260/280 nm ratios. The cRNA probes from wild-type and *acl5-1* seedlings were labeled with Cy3 and Cy5 dyes, respectively, with a Low RNA Fluorescent Linear Amplification Kit (Agilent). After hybridization in Agilent SureHyb chambers, slides were washed according to the manufacturer's instructions and scanned using an Agilent G2505C Microarray Scanner. The scanned images were processed with Feature Extraction software 10.10 (Agilent). The microarray data were submitted to the ArrayExpress database (http://www.ebi.ac.uk/arrayexpress) under accession number E-MTAB-2333. The data were subjected to LOWESS normalization and statistical significance was tested by an unpaired *t*-test with GeneSpring GX software (Agilent). *P* < 0.05 were considered to be statistically significant. Gene ontology (GO) analysis was performed using the AgriGO online tool (Zhou et al., 2010) (http://bioinfo.cau.edu.cn/agriGO/analysis.php).

### Microscopy

For observation of xylem development, seedlings were fixed in ethanol/acetic acid (6:1) overnight, incubated twice in 100% ethanol for 30 min, once in 70% ethanol for 30 min, and cleared with chloral hydrate:glycerol:water mixture (8:1:2, w:v:v) overnight (Kakehi et al., 2010). Samples were mounted on a microscope slide and examined under differential interference contrast microscopy.

### Polyamine analysis

Polyamines were extracted by grinding 0.5 g fresh weight of the aerial parts of agar-grown seedlings in liquid nitrogen, suspending the powder in 2.5 ml of 5% (w/v) perchloric acid, and incubating on ice for 1 h. After centrifugation at 15,000 g for 30 min, 2 ml of the supernatant was filtered through syringe filter of 0.2 μm pore size, neutralized by 1 ml 2 N NaOH, incubated with 10 μl of benzoyl chloride for 20 min, mixed with 2 ml saturated NaCl and then added to 2 ml of diethyl ether. After vigorous shaking and centrifugation at 3000 g for 10 min, the ether layer was evaporated under vacuum and the residue was suspended in 50 μl of methanol. The benzoylated sample was analyzed using reverse phase HPLC system equipped with TSKgel ODS-80Ts column (Toso, Tokyo, Japan). The elution was performed with 42% (v/v) acetonitrile at a flow rate of 0.5 ml/min for 50 min and monitored by measuring its UV absorbance at 254 nm.

## Results

### Microarray analysis of *acl5-1*

To examine the effect of thermospermine deficiency on gene expression profiles in *Arabidopsis*, we performed microarray experiments with RNA samples from 7-day-old *acl5-1* and wild-type seedlings. The results from three independent experiments identified 173 genes whose transcript level was reproducibly increased more than two-fold in *acl5-1* (Supplemental Table 1). GO analysis of these 173 genes revealed significant enrichment of the genes encoding cell wall-related proteins (Supplemental Table 2). These include enzymes involved in cell-wall carbohydrate metabolism such as glycosyl hydrolase family proteins, laccases, pectate lyases, and peroxidases. Representative genes are listed in Table 3. Expression of the genes related to proteolysis such as those encoding serine proteases of the subtilisin family (i.e., *SBT1.1* and *SBT5.2*) and cysteine peptidases (*XCP1* and *XCP2*) were also increased in *acl5-1* seedlings. *XCP1* has been implicated in the developmental cell death associated with lignified xylem vessel differentiation (Funk et al., 2002). The data also confirmed previous findings that *ACL5* and *SAMDC4/BUD2* are up-regulated in *acl5-1*, indicating a negative feedback regulation of these polyamine biosynthetic genes by thermospermine (Kakehi et al., 2008). *SAMDC4/BUD2* is one of the four genes encoding S-adenosylmethionine decarboxylase, which produces decarboxylated S-adenosylmethionine for the synthesis of higher polyamines (Ge et al., 2006), and has been implicated specifically in the synthesis of thermospermine (Kakehi et al., 2010). With regard to transcription factors, members of the NAC-domain protein gene family (*VND1*, *VND2*, *VND6*, *VND7*, and *XND1*), and those of the class III homeodomain leucine-zipper (HD-ZIP III) gene family (*ATHB8*, *PHB*, and *CNA*), which are all involved in the regulation of vascular differentiation, were up-regulated in *acl5-1*. This is consistent with previous reports that showed up-regulation of the members of these gene families in *acl5* mutants (Imai et al., 2006; Kakehi et al., 2008; Muñiz et al., 2008). The mutant also showed increased transcript levels of *MYB46*, a key player in the regulation of secondary wall biosynthesis in fibers and vessels in stems (Zhong et al., 2007). We further found that the transcript level of *MONOPTEROS* (*MP*)/*AUXIN RESPONSE FACTOR5* (*ARF5*), which encodes an auxin-responsive transcription factor (Hardtke and Berleth, 1998), was higher in *acl5-1* than in the wild type. In addition, *TARGET OF MP 5* (*TMO5*), which encodes a bHLH transcription factor and has been shown to be a direct target of *MP/ARF5* (Schlereth et al., 2010), and *TMO5-LIKE1* (*TMO5L1*) were also up-regulated in *acl5-1*. As for signaling molecules, *PHLOEM INTERCALATED WITH XYLEM/TDIF RECEPTOR* (*PXY/TDR*), which encodes a receptor kinase and acts in the promotion of cambial cell proliferation and in the suppression of its differentiation into xylem (Fisher and Turner, 2007; Hirakawa et al., 2008), was up-regulated in *acl5-1*.

**Table 3:** **List of representative genes up-regulated in *acl5-1* seedlings**.

**Functional category**	**AGI Code**	**Annotation**	**Fold change ± SE**
Polyamine metabolism	At5g18930	S-adenosylmethionine decarboxylase 4 (SAMDC4/BUD2)	21.3±2.4
	At5g19530	Thermospermine synthase/ACAULIS 5 (ACL5)	11.6±1.4
Cell-wall metabolism	At3g25050	Xyloglucan endotransglycosylase/hyrdolase 3 (XTH3)	38.5±3.4
	At2g22620	Rhamnogalacturonate lyase	17.2±5.5
	At1g77790	Glycosyl hydrolase family 17/Endo-1,3-beta-glucanase	14.7±2.7
	At5g08370	Alpha-galactosidase 2 (AGAL2)	13.5±3.6
	At2g46570	Laccase 6 (LAC6)	12.5±2.4
	At3g19620	Glycosyl hydrolase family 3/Beta-xylosidase A	10.2±1.4
	At2g46760	D-arabinono-1,4-lactone oxidase family protein	7.8±2.2
	At1g56710	Glycosyl hydrolase family 28/Polygalacturonase	7.4±1.8
	At3g27400	Pectate lyase	7.0±0.6
	At5g51890	Peroxidase involved in TE lignification	5.9±0.9
	At2g34790	FAD-binding domain-containing protein (MEE23/EDA28)	5.2±1.4
	At5g17420	Cellulose synthase/IRREGULAR XYLEM 3 (IRX3)	5.1±0.8
Proteolysis	At5g22860	Serine carboxypeptidase S28	25.0±2.5
	At1g01900	Subtilisin-like serine protease (SBT1.1)	9.7±2.4
	At1g20160	Subtilisin-like serine protease (SBT5.2)	8.7±0.6
	At2g04160	Subtilisin-like protease (AIR3)	6.0±1.3
	At4g35350	Xylem cysteine endopeptidase 1 (XCP1)	5.4±0.5
	At1g20850	Xylem cysteine endopeptidase 2 (XCP2)	3.9±1.1
Transcription	At2g18060	Vascular-related NAC domain protein 1 (VND1)	16.4±4.0
	At4g36160	Vascular-related NAC domain protein 2 (VND2)	12.0±1.9
	At5g62380	Vascular-related NAC domain protein 6 (VND6)	5.9±0.7
	At4g32880	Class III homeodomain-leucine zipper protein (ATHB8)	5.8±1.5
	At1g68810	Basic helix-loop-helix (bHLH) family protein (TMO5L1)	5.7±1.8
	At5g64530	Xylem NAC domain 1 (XND1)	5.5±1.3
	At1g53160	Squamosa promoter-binding protein-like 4 (SPL4)	5.5±2.5
	At1g19850	Auxin response factor 5/MONOPTEROS (MP/ARF5)	5.1±2.1
	At5g12870	Myb protein (MYB46)	4.7±0.7
	At4g13480	Myb protein (MYB79)	4.4±1.0
	At1g71930	Vascular-related NAC domain protein 7 (VND7)	4.2±1.4
	At3g25710	Basic helix-loop-helix (bHLH) family protein (TMO5)	4.0±0.9
	At2g34710	Class III homeodomain-leucine zipper protein (PHB)	3.8±1.3
	At1g52150	Class III homeodomain-leucine zipper protein (CNA/ATHB15)	2.8±1.2
Signal transduction	At5g61480	Leucine-rich repeat transmembrane kinase (PXY/TDR)	5.0±1.9
	At2g01950	Leucine-rich repeat transmembrane kinase (BRL2/VH1)	3.0±1.1
Hormone-related	At1g77110	Auxin transport protein/PIN-FORMED 6 (PIN6)	10.7±2.0
	At5g55250	IAA carboxylmethyltransferase (IAMT1)	5.7±1.8
	At3g53450	Cytokinine-activating enzyme/LONELY GUY 4 (LOG4)	5.0±1.8
	At4g13260	Flavin-containing monooxygenase/YUCCA2 (YUC2)	4.8±0.6
	At2g26700	Protein kinase/PINOID2 (PID2)	4.0±1.5
	At1g73590	Auxin transport protein/PIN-FORMED 1 (PIN1)	3.0±0.7
Transport	At4g02700	Sulfate transporter (SULTR3;2)	6.8±2.2
	At1g77380	Amino acid permease (AAP3)	4.4±1.0
	At2g21050	Amino acid permease/LIKE AUXIN RESISTANT 2 (LAX2)	3.7±1.2

On the other hand, our results revealed only 14 genes that were down-regulated more than two-fold in *acl5-1* (Supplemental Table 3). Only two genes, *IRT1*, and *ZIP8*, which are highly homologous to each other and code for a metal transporter (Vert et al., 2002), showed a more than five-fold reduced expression in the mutant.

### Response of the selected genes to thermospermine

**Figure 1:**
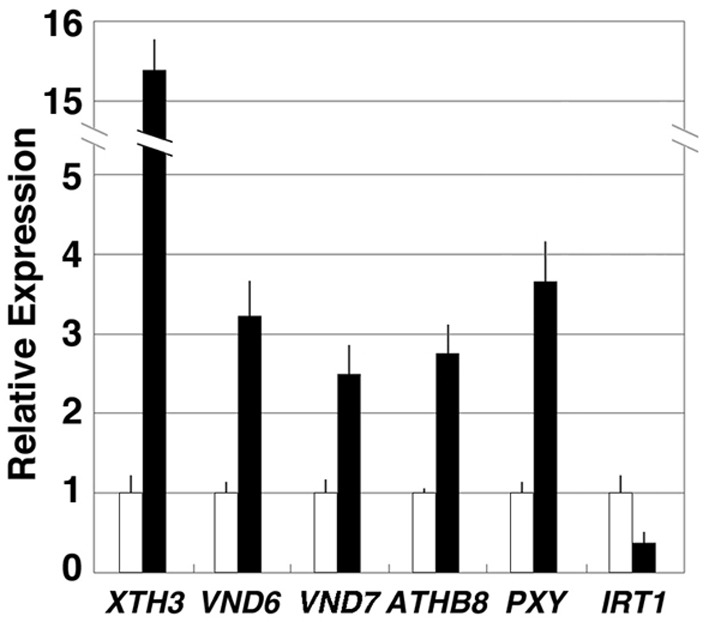
**Expression levels of selected genes altered in *acl5-1***. Total RNA was prepared from 7-day-old wild-type (Col-0) and *acl5-1* seedlings, and analyzed by quantitative real-time RT-PCR. All transcript levels in *acl5-1* (black bars) are relative to those in the wild type (white bars). *ACTIN8* transcripts were used as internal control. Error bars represent the SE (*n* = 3).

*XYLOGLUCAN ENDOTRANSGLYCOSYLASE/HYDROLASE3 (XTH3)*, *VND6*, *VND7*, *ATHB8*, *PXY/TDR*, and *IRT1* were selected for further analysis and their transcript levels were validated by real-time RT-PCR. Our results confirmed that all of these genes except *IRT1* were up-regulated in *acl5-1* seedlings compared to wild-type seedlings (Figure 1).

**Figure 2:**
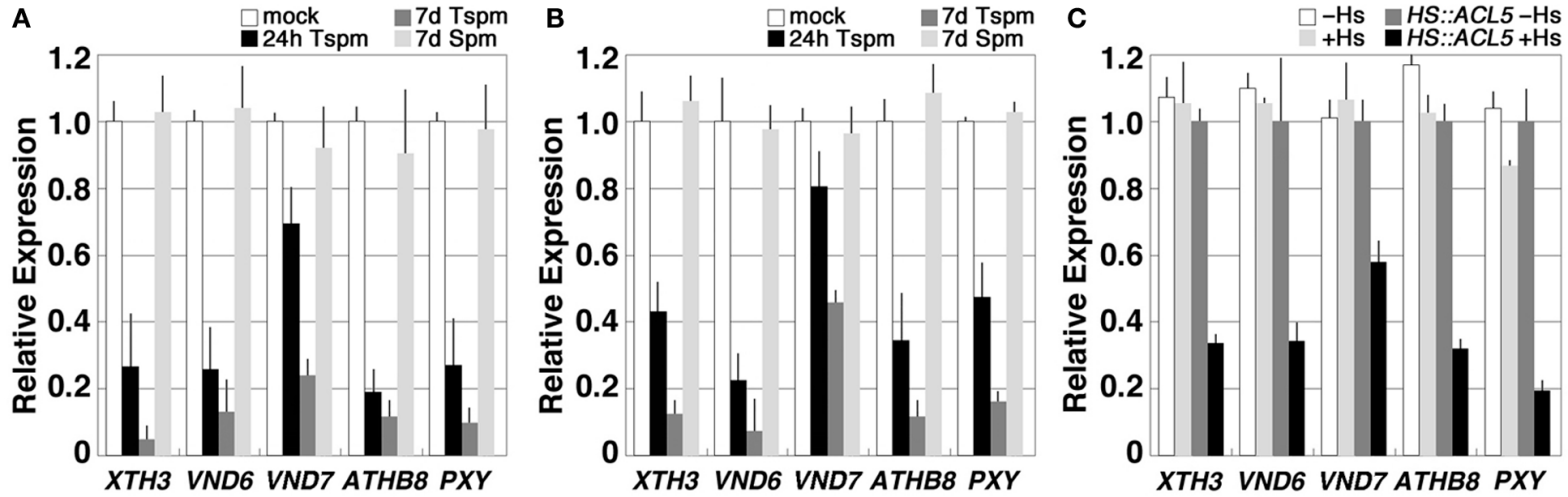
**Thermospermine down-regulates the genes that are up-regulated in *acl5-1*. (A,B)** Effect of exogenous thermospermine and spermine in *acl5-1* **(A)** and wild-type **(B)** seedlings on the expression of the genes up-regulated in *acl5-1*. Transcript levels in seedlings grown for 7 days in MS solutions and incubated for 24 h in MS plus 100 μM thermospermine (black bars) and those in seedlings grown for 7 days in MS solutions supplemented with thermospermine (dark gray bars) or spermine (light gray bars) are shown relative to those in seedlings grown for 7 days in MS solutions with no polyamines (white bars). **(C)** Effect of endogenously-induced thermospermine on the expression of the genes up-regulated in *acl5-1*. Transcript levels in *acl5-1* seedlings heat-shocked at 37°C for 2 h followed by 24-h culture at 22°C (light gray bars), those in transgenic *acl5-1* seedlings carrying the *HS-ACL5* construct untreated (dark gray bars) and those in transgenic *acl5-1* seedlings carrying the *HS-ACL5* construct heat-shocked at 37°C for 2 h followed by 24-h culture at 22°C (black bars), are shown relative to those in *acl5-1* seedlings untreated (white bars). Error bars represent the SE (*n* = 3).

We then examined the effect of exogenous application of thermospermine on the expression of these up-regulated genes in *acl5-1*. Treatment of 7-day-old *acl5-1* seedlings with 100 μM thermospermine for 24 h led to a marked reduction in the transcript levels of these genes except for *VND7* (Figure 2A). When grown in MS solutions with 100 μM thermospermine for 7 days, the *acl5-1* seedlings showed a drastic reduction in the transcript levels of all of the genes tested, compared to those grown without thermospermine and those grown with 100 μM spermine (Figure 2A). Similar responses of these genes were also observed in wild-type seedlings (Figure 2B).

The effect of thermospermine on gene expression was also examined using transgenic *acl5-1* plants carrying a heat-shock inducible *ACL5* (*HS-ACL5*) cDNA. A previous study has shown that the dwarf phenotype of *acl5-1* is complemented in a heat-shock dependent manner by using this transgenic system (Hanzawa et al., 2000). Similar to the case of exogenous treatment for 24 h, transcript levels of all of the genes examined showed a reduction at 24 h after heat-shock treatment for 2 h (Figure 2C).

On the other hand, we confirmed that *IRT1* expression was reduced in *acl5-1* seedlings (Figure 1). However, *IRT1* expression was increased neither by treatment with thermospermine in *acl5-1* and wild-type seedlings nor by heat shock in transgenic *acl5-1* plants carrying the *HS-ACL5* (not shown).

### Thermospermine negatively regulates auxin-related genes

**Figure 3:**
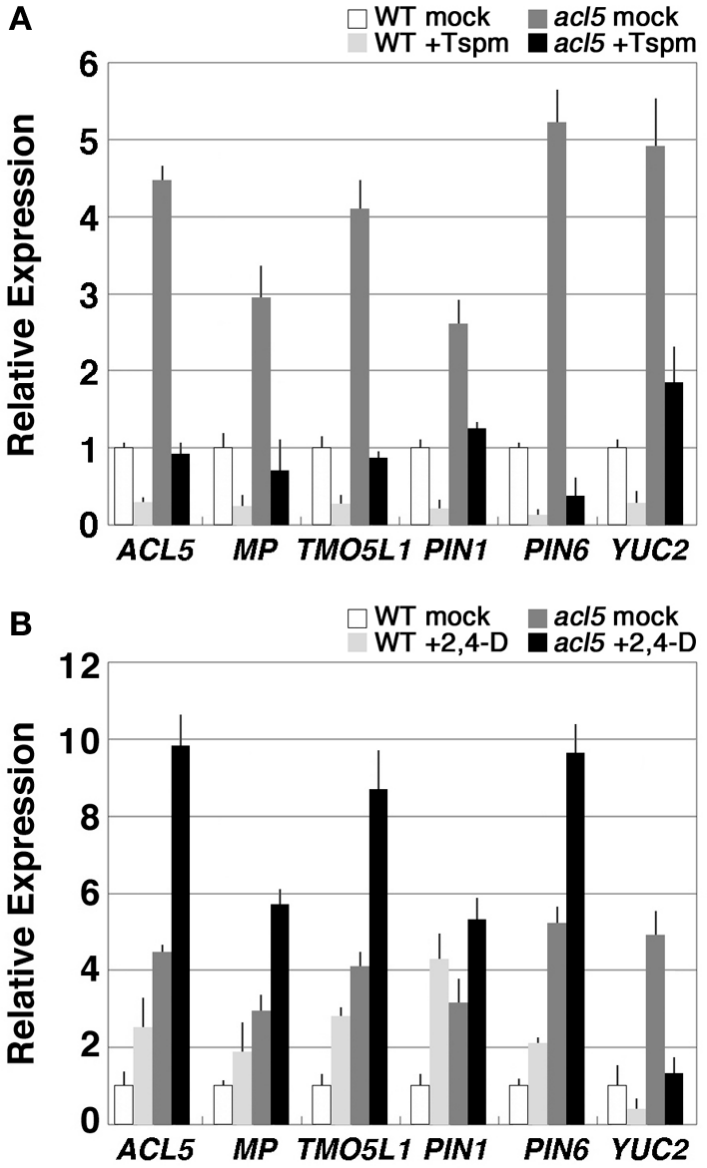
**Thermospermine down-regulates the genes that are involved in auxin biosynthesis, transport, and signaling in *acl5-1*. (A)** Effect of thermospermine on the expression of auxin-related genes. Transcript levels in wild-type seedlings treated for 24 h with 100 μM thermospermine (light gray bars), those in *acl5-1* seedlings treated with mock (dark gray bars) and those in *acl5-1* seedlings treated with thermospermine (black bars), are shown relative to those in wild-type seedlings treated with mock (white bars). **(B)** Effect of 2,4-D on the expression of auxin-related genes. Transcript levels in wild-type seedlings treated for 24 h with 100 μM 2,4-D (light gray bars), those in *acl5-1* seedlings treated with mock (dark gray bars) and those in *acl5-1* seedlings treated with 2,4-D (black bars), are shown relative to those in wild-type seedlings treated with mock (white bars). Error bars represent the SE (*n* = 3).

We have recently found that 2,4-D and its derivatives enhance xylem formation in *acl5-1* but not in the wild type, suggesting repressive control of auxin-induced xylem formation by thermospermine (Yoshimoto et al., 2012). Auxin-induced procambium formation is mediated by *MP/ARF5* (Przemeck et al., 1996; Hardtke and Berleth, 1998), which was listed as an up-regulated gene in *acl5-1* (Table 3). In addition, genes related to auxin biosynthesis and transport such as *PIN-FORMED1* (*PIN1*), *PIN6*, and *YUCCA2* (*YUC2*) were also up-regulated in *acl5-1* (Table 3). *PIN1* and *PIN6* encode an auxin efflux carrier (Petrásek et al., 2006), while *YUC2* encodes a flavin monooxygenase essential for auxin biosynthesis (Cheng et al., 2006). We thus examined the effect of thermospermine on the expression of these genes by real-time RT-PCR. All of these genes showed higher levels of expression in *acl5-1* than in the wild type and were down-regulated by thermospermine in both *acl5-1* and wild-type seedlings (Figure 3A). We also confirmed that transcript levels of all of these genes, except for *YUC2*, were up-regulated by treatment with 100 μM 2,4-D for 24 h in both wild-type and *acl5-1* seedlings (Figure 3B). As reported previously (Hanzawa et al., 2000; Kakehi et al., 2008), *ACL5* expression is down-regulated by thermospermine (Figure 3A) and up-regulated by 2,4-D (Figure 3B). Transcript levels of these genes were also increased in flowering shoots of *acl5-1* compared to those of the wild type, and down-regulated by heat shock in transgenic *acl5-1* plants carrying the *HS-ACL5* (not shown).

**Figure 4:**
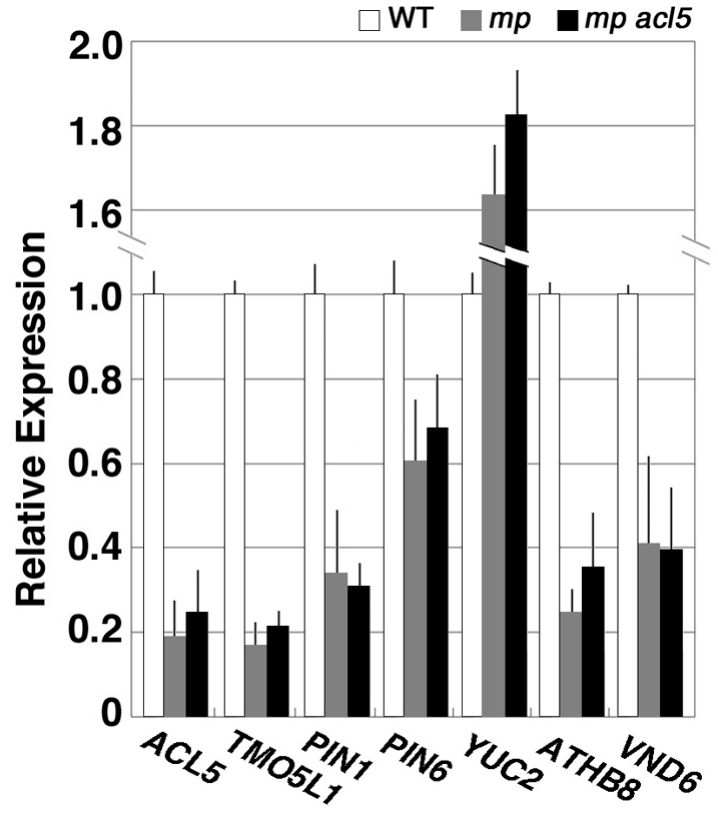
**Expression of thermospermine-responsive genes in the *mp* mutant**. Total RNA was prepared from seedlings of the L*er* wild type (white bars), *mp* (gray bars), and *mp acl5-1* (black bars) grown for 7 days in MS agar plates. All transcript levels are relative to those in wild-type seedlings. Error bars represent the SE (*n* = 3).

We further examined expression of these genes in *mp*, a seedling-lethal allele of *MP/ARF5* that lacks basal body structures such as hypocotyl and root meristem (Berleth and Jürgens, 1993). Transcript levels of all of the auxin-related genes examined except for *YUC2* were reduced in *mp* seedlings and were not restored to wild-type levels in *mp acl5-1* seedlings (Figure 4), which are morphologically indistinguishable from *mp*.

### Artificial tetramines can mimic the effect of thermospermine

**Figure 5:**
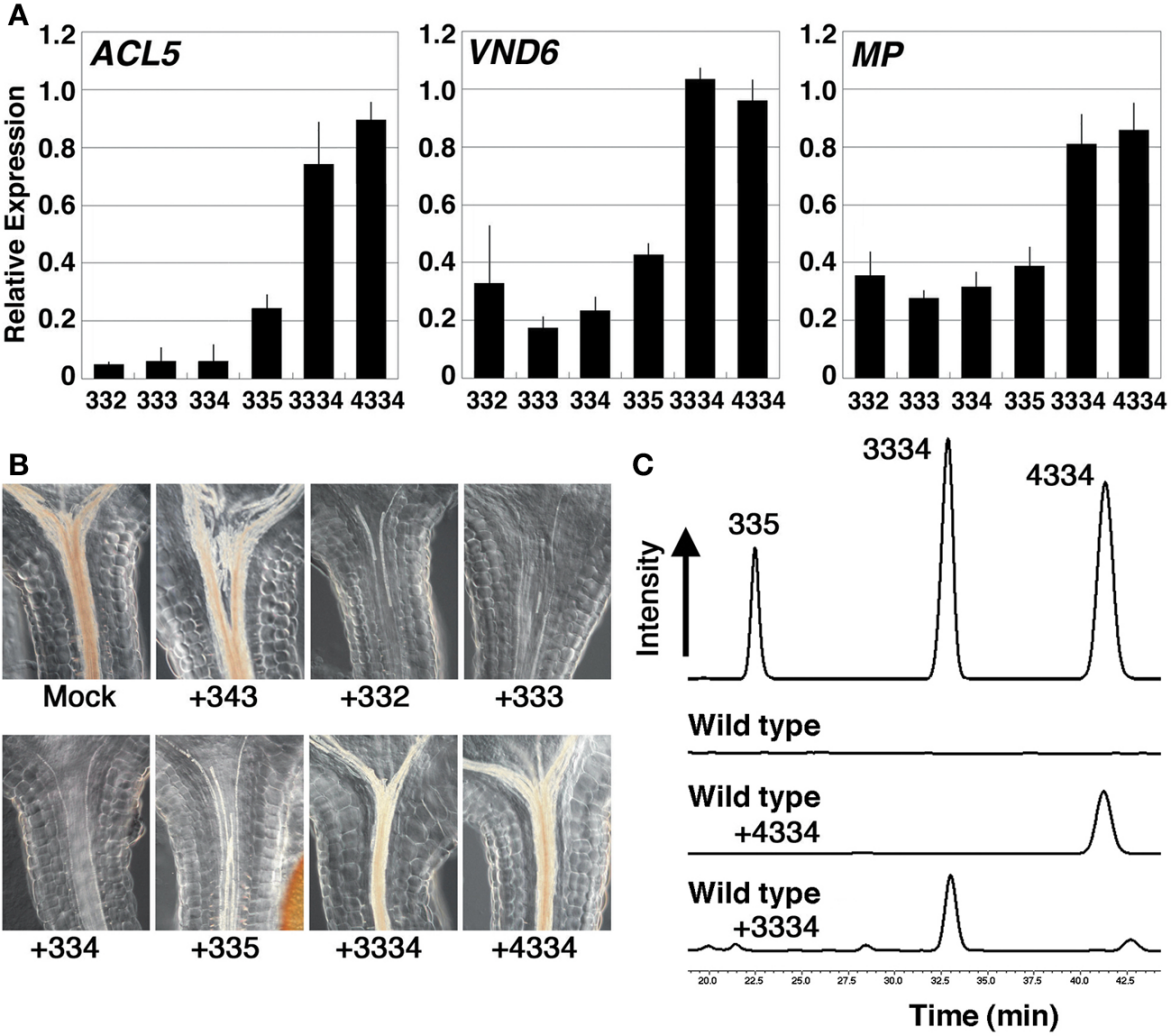
**Effect of polyamines in the *acl5-1 spms-1* double mutant. (A)** Effect of polyamines on the expression of *ACL5*, *VND6*, and *MP*. Total RNA was prepared from *acl5-1 spms-1* seedlings grown for 7 days in MS solutions and treated for 24 h in MS plus 100 μM each polyamine. Numbers correspond to those of the carbon shown in Table 1. All transcript levels are relative to those in mock-treated *acl5-1 spms-1* seedlings. Error bars represent the SE (*n* = 3). **(B)** Effect of polyamines on xylem development. *acl5-1 spms-1* seedlings were grown for 7 days in MS solutions supplemented with 100 μM each polyamine. **(C)** HPLC separations of benzoylated-polyamines from the aerial part of wild-type seedlings. Seedlings were grown for 7 days in MS agar plates supplemented with homocaldopentamine (C3C3C3C4), N,N'-bis(4-aminobutyl)norspermidine (C4C3C3C4) or no polyamines. The elution pattern of C3C3C5, C3C3C3C4 and C4C3C3C4 polyamines is shown above as a reference.

We next examined the effect of 24-h treatment with synthetic polyamines on *ACL5*, *VND6*, and *MP/ARF5* in *acl5-1 spms-1* seedlings, which produce no tetramines. In addition to thermospermine and norspermine, tetramines of 3-3-2 and 3-3-5 (Table 1) significantly repressed the expression of these genes, while homocaldopentamine (3-3-3-4) and the 4-3-3-4 pentamine had little or no effect on their expression (Figure 5A). Effect of these synthetic polyamines on xylem development was also examined under microscopy. The *acl5-1 spms-1* seedlings were grown in the presence of each polyamine for 7 days. As is the case with thermospermine and norspermine (Kakehi et al., 2010), 3-3-2 and 3-3-5 tetramines drastically reduced excess accumulation of lignin but homocaldopentamine and the 4-3-3-4 pentamine had little or no reduction in *acl5-1 spms-1* (Figure 5B). We confirmed that these higher polyamines were absorbed by the root and transported to the shoot by detecting the content of polyamines in the aerial part of the seedlings with HPLC (Figure 5C).

**Figure 6:**
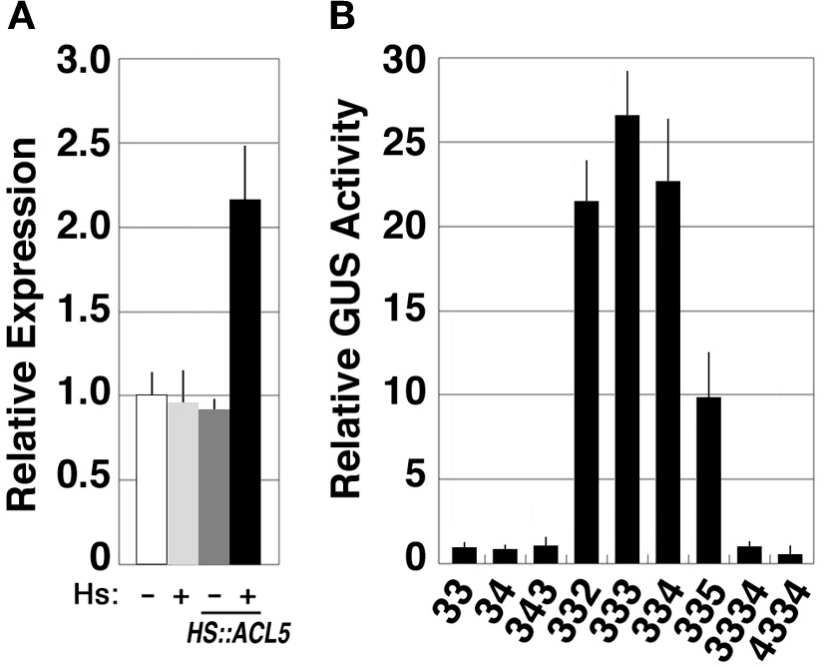
**Responses of *SAC51* to polyamines in *acl5-1*. (A)** Effect of endogenously-induced thermospermine on *SAC51* expression. Transcript levels in *acl5-1* seedlings heat-shocked at 37°C for 2 h followed by 24-h culture at 22°C (light gray bars), those in transgenic *acl5-1* seedlings carrying the *HS-ACL5* construct untreated (dark gray bars) and those heat-shocked at 37°C for 2 h followed by 24-h culture at 22°C (black bars), are shown relative to those in *acl5-1* seedlings untreated (white bars). **(B)** Effect of polyamines on *SAC51-GUS* expression. Transgenic *acl5-1* seedlings carrying the GUS gene fused to the *SAC51* promoter and its 5′ leader sequence were grown for 7 days in MS solutions supplemented with each polyamine. Numbers correspond to those of the carbon shown in Table 1. The activity is shown relative to that of mock-treated seedlings. Error bars represent the SE (*n* = 3).

Although our microarray experiments did not identify *SAC51* as a gene down-regulated in *acl5-1*, a previous study revealed that the *SAC51* transcript level is increased by thermospermine probably at least in part due to its stabilization (Kakehi et al., 2008). Transgenic *acl5-1* seedlings carrying the *HS-ACL5* construct described above showed an approximately 2-fold increase in the *SAC51* transcript level at 24 h after heat-shock treatment for 2 h (Figure 6A). Furthermore, the GUS reporter activity under the control of the *SAC51* promoter and its 5′ leader sequence is increased by treatment for 24 h with thermospermine and norspermine in transgenic *acl5-1* seedlings (Kakehi et al., 2010). We therefore examined the continuing effect of synthetic polyamines on *SAC51*. Transgenic *acl5-1* seedlings carrying the GUS reporter gene under the control of the *SAC51* promoter and its 5′ leader sequence were grown for 7 days in the presence of each polyamine. The results revealed that the GUS activity was increased by thermospermine, norspermine, 3-3-2, and 3-3-5 tetramines, but unaffected by spermidine, norspermidine, spermine, homocaldopentamine, and the 4-3-3-4 pentamine (Figure 6B).

### Tetramines with the 3-3 chain repress lateral root formation

**Figure 7:**
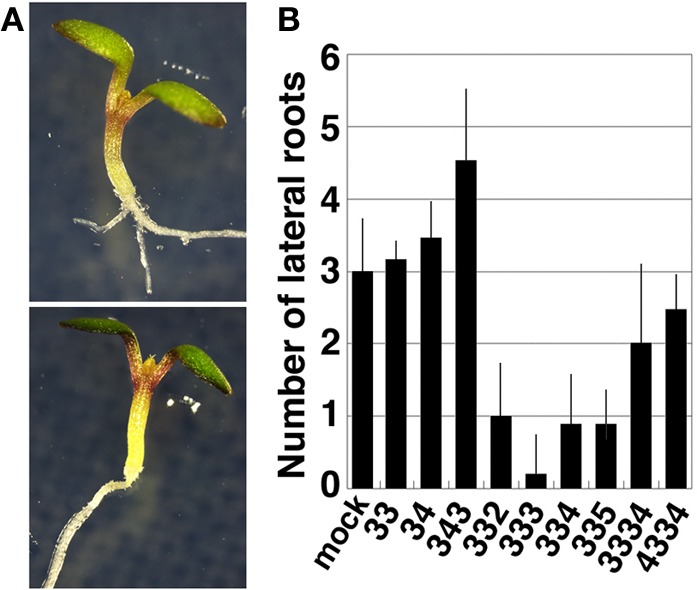
**Effect of polyamines on lateral root formation. (A)** A wild-type seedling grown for 5 days in MS solutions (upper panel) and that supplemented with 100 μM thermospermine (lower panel). **(B)** Number of lateral roots in wild-type seedlings grown for 5 days in MS solutions supplemented with 100 μM each polyamine. Numbers correspond to those of the carbon shown in Table 1. Error bars represent the SE (*n* = 10).

In the course of the study, we found that exogenous treatment with thermospermine has an inhibitory effect on lateral root formation in both wild-type and *acl5-1* seedlings. We then examined whether or not synthetic polyamines have the same effect as thermospermine in wild-type seedlings. The results revealed that lateral root formation was also severely repressed by norspermine, 3-3-2, and 3-3-5 tetramines, but not by spermidine, norspermidine, spermine, homocaldopentamine, and the 4-3-3-4 pentamine (Figure 7).

## Discussion

In bacteria, yeast, and animal cells, effects of polyamines on cell growth were mainly studied at the level of mRNA translation, because polyamines exist to a large extent as RNA-polyamine complexes in cells (Igarashi and Kashiwagi, 2006). A group of genes whose expression is enhanced by polyamines at the level of translation is referred to as a “polyamine modulon.” The polyamine modulon includes transcription factors and kinases that in turn activate gene expression of other proteins (Igarashi and Kashiwagi, 2011). In plants, only few genes have been identified as polyamine-responsive genes. In *Arabidopsis*, spermine up-regulates expression of transcription factor genes such as *WRKY40* and *bZIP60*. However, since amine oxidase inhibitors cancel this response, it may be triggered by hydrogen peroxide derived from oxidative degradation of spermine (Mitsuya et al., 2009). On the other hand, thermospermine has been shown to negatively regulate expression of *ACL5* and the members of the HD-ZIP III gene family such as *ATHB8* and *PHB* (Kakehi et al., 2008). In agreement with the phenotype of *acl5-1*, which shows excess differentiation of lignified xylem cells, a number of genes involved in the regulation of xylem differentiation and those involved in secondary cell wall formation were up-regulated in *acl5-1* seedlings and down-regulated by exogenously supplied and endogenously induced thermospermine. Amongst them was included *MP/ARF5*. *MP/ARF5* encodes a transcription factor of the ARF family, which is activated by auxin-dependent degradation of its interacting repressor, *BODENLOS/INDOLE ACETIC ACID-INDUCED PROTEIN 12* (*BDL*/*IAA12*), and acts as a master regulator for the establishment of vascular and body patterns in embryonic and post-embryonic development (Berleth and Jürgens, 1993; Przemeck et al., 1996; Hardtke and Berleth, 1998; Mattsson et al., 2003; Hardtke et al., 2004; Weijers et al., 2005). Mutations in *MP/ARF5* interfere with the formation of vascular strands and with the initiation of the body axis in the early embryo (Przemeck et al., 1996). In recent studies, such transcription factors as *ATHB8* and *TMO5* have been identified as a direct target of *MP/ARF5* (Donner et al., 2009; Schlereth et al., 2010). Thus, auxin-induced expression of *ATHB8* found in earlier studies (Baima et al., 1995) appears to be mediated by *MP/ARF5*. *MP/ARF5* is also suggested to play a pivotal role in a positive feedback loop for auxin canalization by stimulating expression of *PIN1* (Wenzel et al., 2007). We confirmed that transcript levels of these genes were also increased in *acl5-1* and decreased by thermospermine. This is possibly due to the effect of *MP/ARF5* expression. In fact, they were reduced in *mp* seedlings. Based on these results together with previous phenotypic analyses of *acl5* alleles (Hanzawa et al., 1997; Clay and Nelson, 2005; Imai et al., 2006; Kakehi et al., 2008; Muñiz et al., 2008) and a chemical biology approach revealing that that 2,4-D derivatives enhance the excess xylem phenotype of *acl5-1* but have no effect on wild-type xylems (Yoshimoto et al., 2012), it is possible that thermospermine plays an opposing role to auxin in xylem differentiation through negative regulation of the expression of *MP/ARF5*.

On the other hand, a previous study has shown that knockdown of the HD-ZIP III genes by transgenic overexpression of microRNA *miR165*, which targets all the five HD-ZIP III genes in *Arabidopsis*, results in the reduced expression of *ACL5* and a subset of the genes related to vascular formation including *XCP1*, *XCP2* and *IRX3* (Zhou et al., 2007). These transcript levels were increased along with the *acl5-1* mutant transcript level in *acl5-1* seedlings (Table 3). Quadruple mutations of the five HD-ZIP III genes cause the lack of metaxylem development and little or no expression of *ACL5* in the root tissue (Carlsbecker et al., 2010). These results indicate that the HD-ZIP III transcription factors directly or indirectly activate the *ACL5* expression. It is also noted that *ACL5* expression was reduced in *mp* (Figure 4), suggesting that *ACL5* expression follows the onset of vascular formation by *MP*. Although the *ATHB8* expression is under the direct control of *MP/ARF5*, *ATHB8* is postulated to play a role in stabilizing procambium precursor cells to narrow regions against perturbations in auxin flow (Donner et al., 2009). Thus, the role of *ATHB8* might be virtually performed by thermospermine. In vascular formation, undifferentiated stem cells become procambium precursor cells, procambial cells, xylem precursor cells, and eventually three types of xylem cells: vessels, fibers, and parenchyma cells (Lehesranta et al., 2010). While *MP/ARF5* and *ATHB8* act in the early steps of this process, downstream thermospermine might participate in a negative feedback loop that fine-tunes *MP* expression. A previous study suggests that *ACL5* functions in preventing xylem precursors from premature cell death before secondary cell wall formation based on the observation that metaxylem vessels and xylem fibers are absent in *acl5* mutants (Muñiz et al., 2008). Taking into account the vast effect of thermospermine on the expression of such key regulators as *MP/ARF5*, HD-ZIP III, and *VND* genes, it is apparent that thermospermine plays a major inhibitory role against auxin during the process of continuing vascular formation.

Interestingly, we found that exogenous thermospermine strongly represses lateral root formation in wild-type seedlings. Since auxin controls lateral root development through multiple auxin-signaling modules, among which MP/ARF5 is one of major regulators (Lavenus et al., 2013), the inhibitory effect of thermospermine on lateral root formation may also be attributed to the reduced expression of auxin-related genes.

Our microarray study did not identify the regulatory genes whose expression is up-regulated by thermospermine. Given the fact that polyamines have enhancing effects on translation of specific mRNAs, a bHLH transcription factor gene *SAC51* whose translation is enhanced by thermospermine is a most probable candidate for a mediator of thermospermine signaling that negatively regulates expression of *MP/ARF5* and/or other regulatory genes for xylem development. The *Arabidopsis* genome has three genes homologous to *SAC51*, all of which contain conserved multiple uORFs within their long 5′ leader regions. These transcription factors might also be up-regulated at the level of translation by thermospermine. On the other hand, two metal transporter genes, *IRT1*, and *ZIP8*, showed reduced expression in *acl5-1* mutants. So far, the causal relationship between thermospermine deficiency and the function of these metal transporters remains unknown and needs further investigation.

The results of the experiments using artificial polyamines revealed that, in addition to norspermine, 3-3-2 and 3-3-5 tetramines are also potent substitutes for thermospermine. Although the efficiency of import and/or transport of polyamines may depend on their structure and size, we confirmed that linear pentamines were absorbed by the root and transported to the shoot but were not able to replace thermospermine in terms of cellular functions. Thus, we suggest that the tetramines containing the core structure of 3-3 are biologically active and they may be useful as novel plant growth regulators against auxin for the control of xylem differentiation and lateral root formation. Since *ACL5* orthologs are present in unicellular algaes (Knott et al., 2007), the original role of thermospermine may be unrelated to cell differentiation in multicellular organisms. It is of interest to speculate that, while retaining the mode of action at the molecular level, thermospermine may have been recruited as a part of a negative feedback control in auxin-induced proliferation of xylem cells, which are programmed to die, during the evolution of vascular systems in land plants. Future work should be done with non-vascular plants to address the original function of thermospermine.

### Conflict of interest statement

The authors declare that the research was conducted in the absence of any commercial or financial relationships that could be construed as a potential conflict of interest.

## Supplementary material

Supplemental Table 1 **List of the genes up-regulated in *acl5-1* seedlings**.

Supplemental Table 2 **GO analysis of the gene up-regulated in *acl5-1* seedlings**.

Supplemental Table 3 **List of the genes down-regulated in *acl5-1* seedlings**.
