# Supplementary material for: Thermospermine modulates expression of auxin-related genes in Arabidopsis
Source: Front Plant Sci. 2014 Mar 14;5:94. doi: 10.3389/fpls.2014.00094 (PMC3953664; doi:10.3389/fpls.2014.00094)
Supplement: Supplemental Table 1 — List of the genes up-regulated in acl5-1 seedlings. [file DataSheet1.DOCX]

**Supplemental Table 1** List of the genes up-regulated in *acl5-1* seedlings

|  | AGI code | Annotation | Fold change | *P* |
| --- | --- | --- | --- | --- |
| 1 | At3g25050 | Xyloglucan endotransglycosylase / hydrolase (XTH3) | 38.48 | 3.86E-03 |
| 2 | At5g22860 | Serine carboxypeptidase S28 | 24.78 | 5.85E-03 |
| 3 | At5g18930 | S-adenosylmethionine decarboxylase (SAMDC4/BUD2) | 21.28 | 1.48E-03 |
| 4 | At2g34700 | Pollen Ole e 1 allergen and extensin family protein | 19.02 | 1.62E-03 |
| 5 | At2g22620 | Rhamnogalacturonate lyase | 17.17 | 1.18E-02 |
| 6 | At3g51030 | Thioredoxin H-type 1 (TRX-H-1) | 16.77 | 9.18E-03 |
| 7 | At2g18060 | Vascular-related NAC domain protein (VND1) | 16.30 | 2.16E-03 |
| 8 | At1g77790 | Glycosyl hydrolase family 17 / Endo-1,3-beta-glucanase | 14.56 | 1.08E-02 |
| 9 | At3g10080 | RmlC-like cupins superfamily protein | 13.76 | 6.49E-03 |
| 10 | At5g08370 | Alpha-galactosidase (AGAL2) | 13.55 | 7.91E-03 |
| 11 | At2g46570 | Laccase (LAC6) | 12.48 | 1.08E-03 |
| 12 | At2g29440 | Glutathione S-transferase 24 (TAU6/GST24) | 12.22 | 3.84E-03 |
| 13 | At4g36160 | Vascular-related NAC domain protein (VND2) | 12.11 | 4.39E-03 |
| 14 | At5g19530 | Thermospermine synthase / ACAULIS 5 (ACL5) | 11.55 | 5.58E-03 |
| 15 | At3g18280 | Lipid transfer protein (LTP) | 10.90 | 1.23E-03 |
| 16 | At1g77110 | Auxin transport protein / PIN-FORMED 6 (PIN6) | 10.68 | 4.44E-03 |
| 17 | At3g19620 | Glycosyl hydrolase family 3 / Beta-xylosidase A | 10.21 | 3.93E-03 |
| 18 | At5g59360 | Exressed protein | 9.95 | 4.70E-03 |
| 19 | At4g01985 | Exressed protein | 9.82 | 4.09E-03 |
| 20 | At1g01900 | Subtilisin-like serine protease (SBT1.1) | 9.73 | 6.70E-03 |
| 21 | At2g28410 | Exressed protein | 9.71 | 1.48E-02 |
| 22 | At4g24430 | Exressed protein | 8.90 | 3.19E-03 |
| 23 | At1g20160 | Subtilisin-like serine protease (SBT5.2) | 8.73 | 4.01E-03 |
| 24 | At2g43120 | RmlC-like cupins superfamily protein | 8.25 | 1.38E-03 |
| 25 | At2g46760 | D-arabinono-1,4-lactone oxidase family protein | 7.82 | 3.34E-03 |
| 26 | At5g16490 | ROP-interactive CRIB motif-containing protein (RIC4) | 7.70 | 1.07E-03 |
| 27 | At3g48030 | Zinc finger/C3HC4-type RING finger family protein | 7.68 | 5.04E-03 |
| 28 | At5g50090 | Exressed protein | 7.66 | 3.20E-03 |
| 29 | At1g56710 | Glycoside hydrolase family 28 / Polygalacturonase | 7.37 | 1.81E-02 |
| 30 | At2g40200 | Basic helix-loop-helix (bHLH) family protein | 7.29 | 7.52E-03 |
| 31 | At3g27400 | Pectate lyase | 6.99 | 3.09E-03 |
| 32 | At1g23340 | Exressed protein | 6.96 | 1.59E-02 |
| 33 | At4g02700 | Sulfate transporter (SULTR3;2) | 6.84 | 1.35E-02 |
| 34 | At3g27200 | Cupredoxin superfamily protein | 6.77 | 5.86E-03 |
| 35 | At5g50790 | Nodulin MtN3 family protein | 6.74 | 1.53E-02 |
| 36 | At1g14190 | Glucose-methanol-choline (GMC) oxidoreductase family protein | 6.57 | 6.44E-03 |
| 37 | At5g07800 | Flavin-binding monooxygenase family protein | 6.52 | 6.09E-03 |
| 38 | At2g37870 | Lipid transfer protein (LTP) | 6.44 | 1.72E-02 |
| 39 | At3g47510 | Exressed protein | 6.39 | 3.18E-03 |
| 40 | At5g59845 | Gibberellin-regulated family protein | 6.26 | 1.11E-02 |
| 41 | At3g26125 | Cytochrome P450 (CYP86C2) | 6.12 | 1.23E-02 |
| 42 | At2g04160 | AUXIN-INDUCED IN ROOT CULTURES 3 (AIR3) | 6.03 | 8.77E-03 |
| 43 | At5g62380 | Vascular-related NAC domain protein (VND6) | 5.89 | 3.00E-03 |
| 44 | At5g51890 | Peroxidase involved in TE lignification | 5.89 | 1.56E-02 |
| 45 | At4g32880 | Class III homeodomain-leucine zipper protein (ATHB8) | 5.86 | 1.69E-02 |
| 46 | At2g04850 | Auxin-responsive family protein | 5.76 | 2.83E-02 |
| 47 | At1g23460 | Glycosyl hydrolase family 28 / Polygalacturonase | 5.71 | 3.95E-02 |
| 48 | At5g55250 | IAA carboxylmethyltransferase (IAMT1) | 5.69 | 4.98E-02 |
| 49 | At1g68810 | Basic helix-loop-helix (bHLH) family protein (TMO5L1) | 5.67 | 4.51E-03 |
| 50 | At1g47410 | Exressed protein | 5.65 | 5.56E-03 |
| 51 | At5g64530 | Xylem NAC domain protein (XND1) | 5.52 | 1.49E-02 |
| 52 | At1g53160 | Squamosa promoter-binding protein-like protein (SPL4) | 5.48 | 5.67E-03 |
| 53 | At2g36710 | Pectinesterase | 5.42 | 6.18E-03 |
| 54 | At4g35350 | Xylem cysteine endopeptidase (XCP1) | 5.37 | 1.51E-02 |
| 55 | At2g37090 | Glycosyl transferase family 43 / IRREGULAR XYLEM 9 (IRX9) | 5.33 | 2.54E-02 |
| 56 | At3g51220 | Exressed protein | 5.24 | 5.08E-03 |
| 57 | At1g70470 | Exressed protein | 5.18 | 4.70E-03 |
| 58 | At2g34790 | FAD-binding domain-containing protein (MEE23/EDA28) | 5.17 | 4.62E-03 |
| 59 | At2g41130 | Basic helix-loop-helix (bHLH) family protein | 5.17 | 4.16E-03 |
| 60 | At1g19850 | Auxin response factor / MONOPTEROS (MP/ARF5) | 5.14 | 6.46E-03 |
| 61 | At3g60890 | LITTLE ZIPPER 2 (ZPR2) | 5.14 | 2.59E-02 |
| 62 | At1g67110 | Cytochrome P450 (CYP735A2) / Cytokinin hydroxylase | 5.09 | 4.00E-03 |
| 63 | At5g17420 | Cellulose synthase / IRREGULAR XYLEM 3 (IRX3) | 5.07 | 1.14E-02 |
| 64 | At1g30760 | FAD-binding berberine family protein | 5.04 | 1.13E-07 |
| 65 | At1g11190 | Bifunctional nuclease (BFN1) | 5.03 | 1.35E-02 |
| 66 | At2g44460 | Glycosyl hydrolase family 1 / 6-phospho-beta-galactosidase | 4.97 | 9.62E-03 |
| 67 | At3g53450 | Cytokinine-activating enzyme / LONELY GUY 4 (LOG4) | 4.94 | 4.34E-03 |
| 68 | At5g61480 | Leucine-rich repeat transmembrane kinase (PXY/TDR) | 4.93 | 1.36E-02 |
| 69 | At1g70500 | Glycosyl hydrolase family 28 / Polygalacturonase | 4.91 | 5.27E-03 |
| 70 | At5g03170 | Fasciclin-like arabinogalactan-protein (FLA11) | 4.90 | 1.91E-02 |
| 71 | At1g66810 | Zinc finger (CCCH-type) family protein | 4.90 | 1.43E-02 |
| 72 | At4g38650 | Glycosyl hydrolase family 10 protein | 4.88 | 4.22E-03 |
| 73 | At4g13260 | Flavin-containing monooxygenase / YUCCA2 (YUC2) | 4.84 | 2.37E-02 |
| 74 | At1g64625 | Basic helix-loop-helix (bHLH) family protein | 4.83 | 2.43E-02 |
| 75 | At5g45970 | Rac-like GTP-binding protein (ARAC2/ROP7) | 4.80 | 1.42E-02 |
| 76 | At4g08160 | Glycosyl hydrolase family 10 protein / Xylanase | 4.77 | 5.76E-03 |
| 77 | At3g51380 | Calmodulin-binding family protein / IQ-domain 20 (IQD20) | 4.77 | 8.47E-03 |
| 78 | At1g32100 | Pinoresinol reductase involved in lignan biosynthesis (PRR1) | 4.76 | 5.01E-03 |
| 79 | At2g33530 | Serine carboxypeptidase S10 (SCPL46) | 4.74 | 4.26E-03 |
| 80 | At1g79620 | Leucine-rich repeat transmembrane kinase | 4.70 | 7.89E-03 |
| 81 | At5g12870 | Myb protein (MYB46) | 4.68 | 5.82E-03 |
| 82 | At3g18660 | GLUCURONIC ACID SUBSTITUTION OF XYLAN 1 (GUX1) | 4.58 | 1.08E-02 |
| 83 | At1g10750 | Exressed protein | 4.54 | 5.58E-03 |
| 84 | At5g01890 | Leucine-rich repeat transmembrane kinase | 4.47 | 2.96E-03 |
| 85 | At1g19540 | NmrA-like negative transcriptional regulator family protein | 4.46 | 3.03E-03 |
| 86 | At5g48920 | TRACHEARY ELEMENT DIFFERENTIATION-RELATED 7 (TED7) | 4.40 | 7.98E-03 |
| 87 | At3g62160 | HXXXD-type acyl-transferase family protein | 4.40 | 5.65E-03 |
| 88 | At1g77380 | Amino acid permease (AAP3) | 4.39 | 9.54E-03 |
| 89 | At4g13480 | Myb protein (MYB79) | 4.38 | 4.71E-03 |
| 90 | At2g28760 | UDP-Xylose synthase (UXS6) | 4.32 | 8.51E-03 |
| 91 | At2g38080 | Laccase / IRREGULAR XYLEM 12 (IRX12/LAC4) | 4.30 | 9.55E-04 |
| 92 | At1g75280 | Isoflavone reductase | 4.29 | 7.76E-05 |
| 93 | At1g09890 | Rhamnogalacturonate lyase family protein | 4.25 | 5.04E-03 |
| 94 | At5g07080 | HXXXD-type acyl-transferase family protein | 4.23 | 1.92E-02 |
| 95 | At1g71930 | Vascular-related NAC domain protein 7 (VND7) | 4.20 | 3.71E-03 |
| 96 | At1g23800 | Mitochondrial aldehyde dehydrogenase (ALDH2B7) | 4.17 | 3.16E-03 |
| 97 | At1g03820 | Exressed protein | 4.16 | 2.61E-03 |
| 98 | At4g39230 | Phenylcoumaran benzylic ether reductase-like protein | 4.16 | 2.65E-03 |
| 99 | At4g23690 | Disease resistance-responsive (dirigent-like protein) family protein | 4.16 | 2.74E-03 |
| 100 | At1g54200 | Exressed protein | 4.15 | 3.43E-03 |
| 101 | At3g52900 | Exressed protein | 4.04 | 1.59E-02 |
| 102 | At1g26820 | Ribonuclease (RNS3) | 4.01 | 1.00E-02 |
| 103 | At3g25710 | TARGET OF MONOPTEROS 5 (TMO5/BHLH32) | 4.01 | 1.17E-02 |
| 104 | At5g18860 | Nucleoside hydrolase (NSH3) | 3.99 | 4.64E-03 |
| 105 | At3g14170 | Exressed protein | 3.97 | 4.75E-03 |
| 106 | At2g26700 | Protein kinase / PINOID2 (PID2) | 3.96 | 3.09E-03 |
| 107 | At2g11810 | Monogalactosyl diacylglycerol synthase (MGD3) | 3.95 | 2.11E-03 |
| 108 | At4g12910 | Serine carboxypeptidase S10 (SCPL20) | 3.91 | 2.08E-02 |
| 109 | At3g11750 | Dihydroneopterin aldolase involved in folate synthesis (FOLB3) | 3.91 | 6.21E-03 |
| 110 | At5g13330 | AP2 domain-containing transcription factor (RAP2.6L) | 3.91 | 2.25E-02 |
| 111 | At1g20850 | Xylem cysteine endopeptidase (XCP2) | 3.90 | 8.29E-03 |
| 112 | At4g33810 | Glycosyl hydrolase family 10 protein | 3.84 | 4.56E-03 |
| 113 | At3g62020 | Germin-like protein (GLP10) | 3.83 | 4.21E-03 |
| 114 | At2g34710 | Class III homeodomain-leucine zipper protein (PHB/ATHB14) | 3.81 | 6.36E-04 |
| 115 | At5g38450 | Cytochrome P450 (CYP735A1) / Cytokinin hydroxylase | 3.80 | 4.89E-03 |
| 116 | At1g80130 | Tetratricopeptide repeat (TPR)-like superfamily protein | 3.74 | 9.50E-04 |
| 117 | At1g27920 | Microtubule-associated protein 65-8 (MAP65-8) | 3.69 | 6.91E-03 |
| 118 | At3g16920 | Glycosyl hydrolase family 19 / Class I chitinase (CTL2) | 3.68 | 1.57E-02 |
| 119 | At4g23500 | Glycosyl hydrolase family 28 / Polygalacturonase | 3.68 | 1.62E-02 |
| 120 | At1g14890 | Invertase/pectin methylesterase inhibitor family protein | 3.68 | 1.67E-02 |
| 121 | At3g53100 | GDSL-like lipase/acylhydrolase superfamily | 3.67 | 1.84E-02 |
| 122 | At2g21050 | Amino acid permease / LIKE AUXIN RESISTANT 2 (LAX2) | 3.66 | 1.86E-02 |
| 123 | At2g28315 | Nucleotide/sugar transporter family protein | 3.66 | 2.53E-02 |
| 124 | At3g24450 | Heavy metal transport/detoxification superfamily protein | 3.65 | 2.28E-02 |
| 125 | At4g19810 | Glycosyl hydrolase family 18 / chitinase | 3.64 | 4.53E-03 |
| 126 | At1g05310 | Pectinesterase family protein | 3.59 | 2.08E-02 |
| 127 | At5g54690 | Galacturonosyltransferase 12 (GAUT12/IRX8) | 3.55 | 1.41E-02 |
| 128 | At1g43790 | TRACHEARY ELEMENT DIFFERENTIATION-RELATED 6 (TED6) | 3.54 | 2.89E-03 |
| 129 | At4g30450 | Glycine-rich protein | 3.47 | 4.67E-03 |
| 130 | At1g75500 | Nodulin MtN21 family protein / WALLS ARE THIN 1 (WAT1) | 3.46 | 1.34E-02 |
| 131 | At5g51920 | Pyridoxal phosphate-dependent transferase superfamily protein | 3.45 | 3.27E-03 |
| 132 | At2g13820 | Lipid transfer protein (LTP) family protein | 3.38 | 3.40E-03 |
| 133 | At4g00230 | Subtilisin-like serine endopeptidase (XSP1) | 3.37 | 3.56E-03 |
| 134 | At4g22590 | Trehalose-6-phosphate phosphatase (TPPG) | 3.32 | 2.00E-02 |
| 135 | At4g36470 | S-adenosyl-L-methionine:carboxyl methyltransferase family protein | 3.28 | 1.52E-02 |
| 136 | At2g41260 | Late-embryogenesis abundant protein (ATM17) | 3.25 | 4.69E-03 |
| 137 | At3g15720 | Glycosyl hydrolase family 28 / Polygalacturonase | 3.24 | 5.23E-03 |
| 138 | At1g47670 | Transmembrane amino acid transporter protein | 3.21 | 2.21E-03 |
| 139 | At2g44300 | Lipid transfer protein (LTP) family protein | 3.11 | 8.58E-04 |
| 140 | At5g50160 | Ferric reductase / FERRIC REDUCTION OXIDASE 8 (FRO8) | 3.08 | 9.78E-04 |
| 141 | At4g34540 | Pinoresinol-lariciresinol reductase-like protein | 3.07 | 1.19E-02 |
| 142 | At1g73590 | Auxin transport protein / PIN-FORMED 1 (PIN1) | 3.02 | 5.56E-03 |
| 143 | At2g01950 | Leucine-rich repeat transmembrane kinase (BRL2/VH1) | 3.00 | 3.35E-03 |
| 144 | At3g51480 | Glutamate receptor family protein (GLR3.6) | 2.98 | 5.23E-03 |
| 145 | At1g27440 | Glucuronoxylan glucuronosyltransferase (GUT2/IRX10) | 2.95 | 2.19E-02 |
| 146 | At5g56460 | Protein kinase | 2.93 | 8.97E-03 |
| 147 | At2g40640 | RING/U-box superfamily protein | 2.89 | 3.21E-03 |
| 148 | At3g05650 | RECEPTOR-LIKE PROTEIN 32 (RLP32) | 2.86 | 5.82E-03 |
| 149 | At1g52150 | Class III homeodomain-leucine zipper protein (CNA/ATHB15) | 2.84 | 5.06E-03 |
| 150 | At1g55210 | Disease resistance-responsive (dirigent-like protein) family protein | 2.83 | 1.28E-02 |
| 151 | At5g07030 | Eukaryotic aspartyl protease family protein | 2.83 | 2.46E-03 |
| 152 | At2g16990 | Major facilitator superfamily protein | 2.82 | 2.34E-03 |
| 153 | At1g80050 | Adenine phosphoribosyltransferase (APT2) | 2.77 | 5.71E-04 |
| 154 | At1g63120 | Serine-type endopeptidase / RHOMBOID-LIKE 2 (RBL2) | 2.76 | 1.07E-03 |
| 155 | At3g22550 | Exressed protein | 2.66 | 3.07E-03 |
| 156 | At1g30440 | Phototropic-responsive NPH3 family protein | 2.64 | 3.86E-03 |
| 157 | At3g48970 | Heavy metal transport/detoxification superfamily protein | 2.59 | 3.54E-03 |
| 158 | At3g17420 | Protein kinase / GLYOXYSOMAL PROTEIN KINASE 1 (GPK1) | 2.56 | 2.21E-03 |
| 159 | At2g43620 | Chitinase family protein | 2.55 | 3.94E-03 |
| 160 | At1g61800 | Glucose6-phosphate/phosphate transporter (GPT2) | 2.53 | 3.81E-03 |
| 161 | At5g10520 | Protein kinase / ROP-BINDING PROTEIN KINASE 1 (RBK1) | 2.52 | 3.87E-03 |
| 162 | At2g28870 | Exressed protein | 2.49 | 1.54E-02 |
| 163 | At2g28660 | Chloroplast-targeted copper chaperone protein | 2.38 | 2.67E-04 |
| 164 | At2g24230 | Leucine-rich repeat transmembrane kinase | 2.38 | 1.08E-03 |
| 165 | At2g44450 | Glycosyl hydrolase family 1 / 6-phospho-beta-galactosidase | 2.18 | 1.08E-02 |
| 166 | At3g21270 | Dof-type zinc finger domain-containing protein (ADOF2) | 2.17 | 9.48E-04 |
| 167 | At2g34060 | Peroxidase | 2.16 | 2.66E-03 |
| 168 | At1g69880 | Thioredoxin H-type 8 (ATH8) | 2.15 | 1.50E-03 |
| 169 | At5g60970 | TCP family transcription factor (TCP5) | 2.14 | 2.17E-03 |
| 170 | At4g34320 | Exressed protein | 2.11 | 1.33E-02 |
| 171 | At2g34070 | TRICHOME BIREFRINGENCE-LIKE 37 (TBL37) | 2.05 | 9.39E-04 |
| 172 | At2g33205 | Serine and sphingolipid biosynthesis protein | 2.04 | 2.96E-03 |
| 173 | At5g67230 | Glycosyl transferase family 43 protein (IRX14-L) | 2.01 | 4.76E-03 |
