# Supplementary material for: Thermospermine modulates expression of auxin-related genes in Arabidopsis
Source: Front Plant Sci. 2014 Mar 14;5:94. doi: 10.3389/fpls.2014.00094 (PMC3953664; doi:10.3389/fpls.2014.00094)
Supplement: Supplemental Table 2 — GO analysis of the gene up-regulated in acl5-1 seedlings. [file DataSheet2.DOCX]

**Supplemental Table 2** GO analysis of the gene up-regulated in *acl5-1* seedlings

| **GO term** | **Ontology** | **Description** | **Number in input list** | **Number in BG/Ref** | **p-value** | **FDR** |
| --- | --- | --- | --- | --- | --- | --- |
| GO:0042546 | P | cell wall biogenesis | 10 | 70 | 1.70E-12 | 5.70E-10 |
| GO:0009834 | P | secondary cell wall biogenesis | 7 | 19 | 2.40E-12 | 5.70E-10 |
| GO:0009832 | P | plant-type cell wall biogenesis | 9 | 58 | 9.80E-12 | 1.60E-09 |
| GO:0010089 | P | xylem development | 5 | 16 | 9.20E-09 | 1.10E-06 |
| GO:0010087 | P | phloem or xylem histogenesis | 6 | 40 | 3.50E-08 | 3.40E-06 |
| GO:0070882 | P | cellular cell wall organization or biogenesis | 10 | 247 | 3.80E-07 | 2.60E-05 |
| GO:0005975 | P | carbohydrate metabolic process | 18 | 866 | 3.60E-07 | 2.60E-05 |
| GO:0044036 | P | cell wall macromolecule metabolic process | 5 | 45 | 2.30E-06 | 0.00014 |
| GO:0003002 | P | regionalization | 6 | 112 | 1.60E-05 | 0.00087 |
| GO:0007389 | P | pattern specification process | 6 | 150 | 8.30E-05 | 0.004 |
| GO:0048513 | P | organ development | 13 | 838 | 0.00023 | 0.0094 |
| GO:0022621 | P | shoot system development | 8 | 358 | 0.00033 | 0.01 |
| GO:0044264 | P | cellular polysaccharide metabolic process | 5 | 127 | 0.00035 | 0.01 |
| GO:0009888 | P | tissue development | 8 | 359 | 0.00034 | 0.01 |
| GO:0048367 | P | shoot development | 8 | 355 | 0.00032 | 0.01 |
| GO:0010016 | P | shoot morphogenesis | 6 | 198 | 0.00037 | 0.011 |
| GO:0044085 | P | cellular component biogenesis | 10 | 571 | 0.00045 | 0.012 |
| GO:0009791 | P | post-embryonic development | 11 | 705 | 0.00063 | 0.016 |
| GO:0005976 | P | polysaccharide metabolic process | 5 | 152 | 0.00078 | 0.019 |
| GO:0032502 | P | developmental process | 23 | 2304 | 0.00095 | 0.022 |
| GO:0032501 | P | multicellular organismal process | 21 | 2094 | 0.0014 | 0.031 |
| GO:0030154 | P | cell differentiation | 7 | 355 | 0.0016 | 0.031 |
| GO:0034637 | P | cellular carbohydrate biosynthetic process | 5 | 177 | 0.0015 | 0.031 |
| GO:0007275 | P | multicellular organismal development | 20 | 2020 | 0.002 | 0.039 |
| GO:0016798 | F | hydrolase activity, acting on glycosyl bonds | 17 | 526 | 1.40E-09 | 2.40E-07 |
| GO:0003824 | F | catalytic activity | 82 | 9638 | 5.90E-06 | 0.00039 |
| GO:0004650 | F | polygalacturonase activity | 5 | 77 | 3.30E-05 | 0.0016 |
| GO:0016787 | F | hydrolase activity | 34 | 3468 | 0.00013 | 0.005 |
| GO:0017171 | F | serine hydrolase activity | 6 | 186 | 0.00027 | 0.0075 |
| GO:0016757 | F | transferase activity, transferring glycosyl groups | 9 | 544 | 0.0012 | 0.031 |
| GO:0070011 | F | peptidase activity, acting on L-amino acid peptides | 9 | 579 | 0.0019 | 0.042 |
| GO:0030312 | C | external encapsulating structure | 13 | 407 | 1.20E-07 | 4.90E-06 |
| GO:0005618 | C | cell wall | 13 | 403 | 1.10E-07 | 4.90E-06 |
| GO:0012505 | C | endomembrane system | 31 | 3416 | 0.00076 | 0.016 |
| GO:0031225 | C | anchored to membrane | 6 | 272 | 0.0019 | 0.031 |
