# Supplementary material for: Thermospermine modulates expression of auxin-related genes in Arabidopsis
Source: Front Plant Sci. 2014 Mar 14;5:94. doi: 10.3389/fpls.2014.00094 (PMC3953664; doi:10.3389/fpls.2014.00094)
Supplement: Supplemental Table 3 — List of the genes down-regulated in acl5-1 seedlings. [file DataSheet3.DOCX]

**Supplemental Table 3**  List of the genes down-regulated in *acl5-1* seedlings

|  | AGI code | Annotation | Fold change | *P* |
| --- | --- | --- | --- | --- |
| 1 | At4g19690 | Iron-regulated transporter (IRT1) | 10.8 | 2.88E-03 |
| 2 | At5g45105 | Zinc transporter (ZIP8) | 8.4 | 4.86E-03 |
| 3 | At3g45160 | Putative membrane lipoprotein | 4.8 | 4.66E-03 |
| 4 | At5g24780 | Vegetative storage protein 1 (VSP1)/HAD superfamily phosphatase | 3.5 | 1.55E-03 |
| 5 | At3g48360 | BTB and TAZ domain protein (BT2) | 3.3 | 2.14E-03 |
| 6 | At3g15450 | Aluminium induced protein with YGL and LRDR motifs | 3.2 | 2.18E-03 |
| 7 | At1g79700 | Integrase-type DNA-binding superfamily protein | 2.9 | 3.34E-03 |
| 8 | At5g24420 | 6-phosphogluconolactonase (PGL5) | 2.9 | 1.23E-03 |
| 9 | At4g35770 | Senescence-associated protein/DARK INDUCIBLE 1 (DIN1/SEN1) | 2.3 | 4.72E-03 |
| 10 | At4g26530 | Aldolase superfamily protein | 2.3 | 4.11E-03 |
| 11 | At3g22740 | Homocysteine S-methyltransferase (HMT-3) | 2.2 | 3.83E-03 |
| 12 | At2g17880 | DnaJ heat shock protein | 2.0 | 2.15E-03 |
| 13 | At1g23390 | Kelch repeat-containing F-box family protein | 2.0 | 4.79E-03 |
| 14 | At1g11450 | Nodulin MtN21/EamA-like transporter family protein | 2.0 | 2.66E-03 |
